# Ultra-wideband, wide angle, asymmetric transmission based chiral metasurface for C and X band applications

**DOI:** 10.1038/s41598-021-91126-1

**Published:** 2021-06-03

**Authors:** Syed Hussain Ali Bokhari, Hammad M. Cheema

**Affiliations:** Research Institute for Microwave and Millimeter-Wave Studies (RIMMS), National University of Sciences and Technology (NUST), Islamabad, 44000 Pakistan

**Keywords:** Electrical and electronic engineering, Applied physics

## Abstract

A multi-layered chiral device manifesting asymmetric transmission (AT) facilitating one-way channeling of electromagnetic (EM) waves, based on the inherent polarization is presented. The designed metasurface depicts a high transmission contrast with an efficiency above 80% for an ultra-wide operational range of 6.3–12.3 GHz, constituting a fractional bandwidth of 64%. As an additional feature, the reported metasurface yields robustness against oblique incidences up to 45$^\circ$ while maintaining high transmission efficiency. This report also introduces a unique analogy of the AT based communication system with logic-gates by formulating its truth-table and logic circuit. Furthermore, new insights of AT magnitude’s dependence to oblique incidences are presented on the account of surface impedance mismatch due to TE and TM polarization with varying incidence angle. Moreover, avoidance of grating lobes and the associated transmission deterioration through utilization of electrically small periodic metasurface is presented. The results have been numerically and practically validated yielding state-of-the-art features. Operating within C and X band, the reported work is an ideal candidate for practical AT applications.

## Introduction

Controlling the flow of electromagnetic (EM) waves has long been intriguing to researchers. The fascination to manipulate EM light has given rise to a number of functionalities such as cross-polarization conversion, EM wave absorption, transmission and reflection etc^1–7^. Amidst these wave operations, it was deemed most challenging to obtain a directional control of EM waves i.e. allowing light to pass in one direction while blocking it in the other^8^. Non-reciprocal devices that took advantage of inherent gyrotropy in some materials were firstly proposed^9^ and required an external magnetic field for the breakage of time reversal symmetry. The need of an external influence made them bulky and hence inapt for low profile optical systems. Non reciprocal metamaterials and metasurfaces were then tailored to obtain the same functionality^10^, however the proposed devices generally suffered from losses due to non-linearities and design complexities.

In comparison, Asymmetric Transmission (AT), a phenomenon firstly discovered by Fedotov in 2006^11^ allows manipulation of EM wave direction while remaining in a reciprocal regime. The reciprocity, in fact facilitates the engineering of simple and efficient devices. Conceptually, when an AT device encounters a TM wave (x-polarized) in -z-direction, it is transmitted as a TE wave (y-polarized). However, a TE wave incident in same direction undergoes high amount of reflection and is not transmitted. Contrarily, behavior of device becomes opposite for the case of illumination from opposite direction i.e. the AT device becomes transparent for TE wave in +z-direction while blocks the TM wave in the same direction. It can thus be observed that the AT device offers different response in the two directions depending upon the incident polarization of light. Since the inception of AT phenomenon, many effective design topologies have been published. This includes efficient bi-layered configurations^12–23^ and tri-layered configurations as well, which take advantage of Fabry Perot-like cavity thereby resulting in broadband AT response^24–30^.

**Figure 1:**
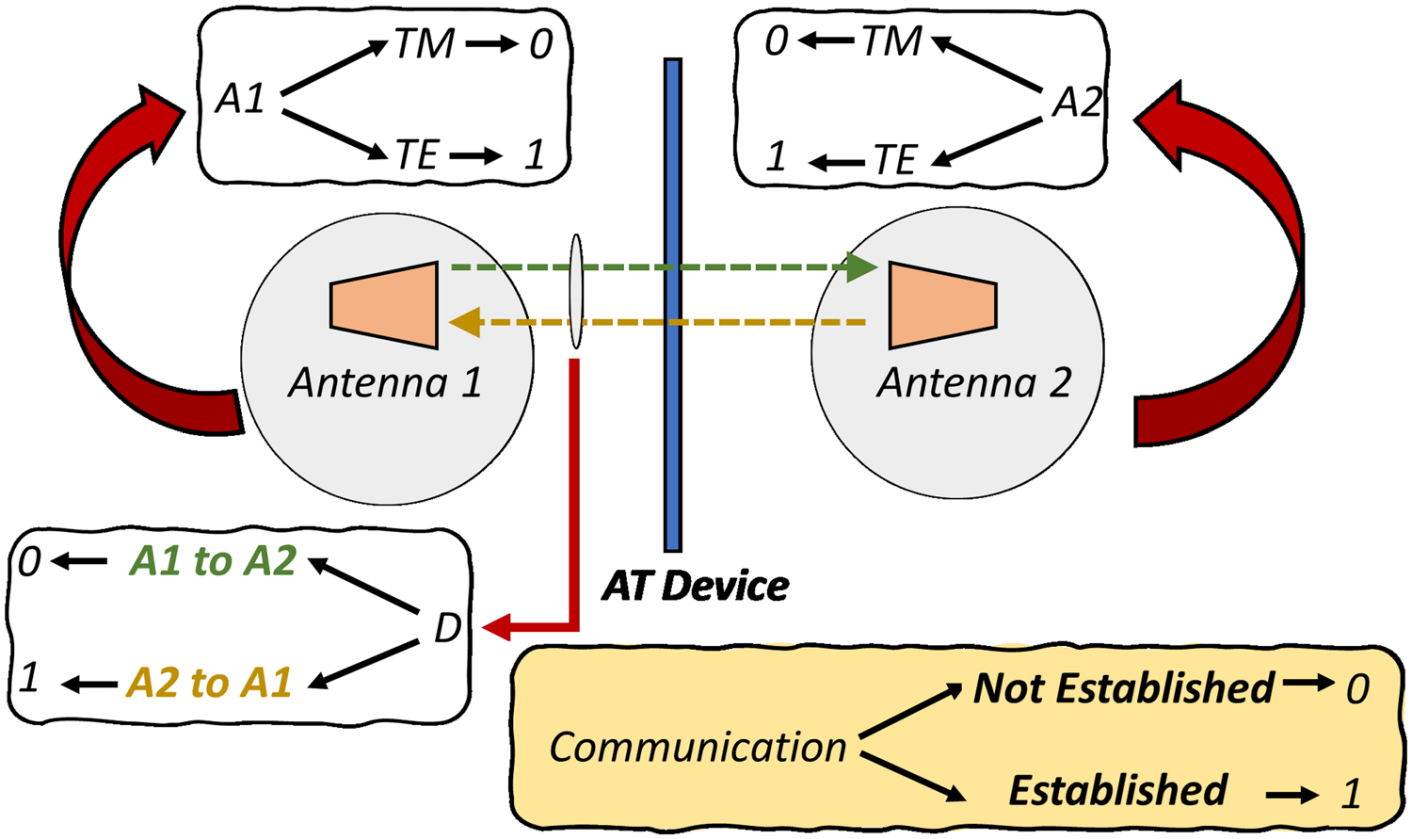
Modeling of logic gates analogy of AT operation.

**Figure 2:**
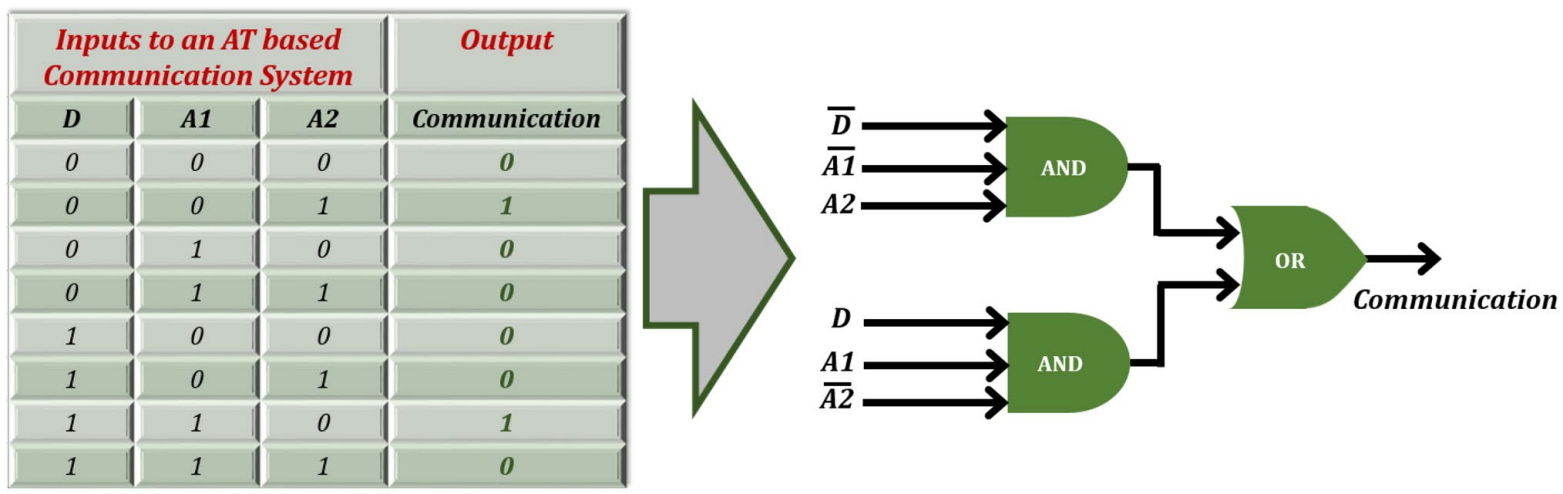
Truth table formulated for the logic gate analogy of AT operation.

The AT phenomenon has been understood through various analogies in the past including electrical models^31^ and physical analyses^12,32^. Adopting a different approach, this paper introduces a new and interesting analogy based on logic-gates to gain deeper insights. Shown in Fig. 1, consider a full/half-duplex communication system having three input entities; Rx/Tx Antenna 1, Tx/Rx Antenna 2 & an AT device between them. Each of these can be characterized by two states 0/1 corresponding to the antenna polarization (TM/TE) and direction of communication (A1 to A2/ A2 to A1). Likewise, the output of such a system can also be characterized as 0/1 depending upon whether a communication channel has been established or not. Next, based on the behavior of a generic AT device, a truth table is devised as shown in Fig. 2. For instance, when both the antennas are of same polarization, no communication is established regardless of direction. However, when the antennas are of different polarization, the establishment of communication becomes dependent on the direction. The formulated table is then processed to form a logic circuit that is fundamentally an OR operation with input being fed from two AND gates whose three inputs D, A1 and A2 are complementary to one another, such that only one of the AND gates can contribute towards enabling the communication channel. It is evident that by switching the direction input to the system, one can control the flow of information between the two antennas. In other words, the communication system becomes direction dependent with respect to the type of polarization incident on the AT device. Such a combinational logic can be utilized as polarization-based control for various modern optical communication applications.

Robustness of any optical device against oblique incidences is an important feature. However, majority of published works have reported device operation at normal incidences alone that generally limits their functionality in practical environments. For instance^33^, presents operation at higher incident angles by the use of PIN diodes thereby making it complex to fabricate. Likewise^34^, only discusses angle of incidence as an extrinsic parameter to tune the structure’s performance^32^, though ensures robust operation up to 30$^{\circ }$, however its magnitude decreases at higher incidences^35,36^ present angularly robust AT devices, however they are severely limited in terms of bandwidth. Authors in their recent work^31^ have also proposed a bi-layered AT device that manifests angular performance up to 60$^{\circ }$, however, its bandwidth though broad, can still be improved to incorporate multi-band operations.

This paper presents a multi-layered chiral device that mimics a Fabry-perot-like cavity resulting in an ultra-broadband AT operation ranging from 6.3 to 12.3 GHz (fractional bandwidth of 64%) with more than 80% transmission efficiency and up to 45$^{\circ }$ angular stability. Furthermore, the dependence of AT magnitude on impedance mismatch with varying incident angles is studied for the first time thereby explaining the inherent sensitivity of AT to oblique incidences. It is also shown that deterioration by grating lobes can be avoided through careful study and design of the miniaturized unit cell.

## Results

### Design of multilayered unit-cell for chiral metasurface

**Figure 3:**
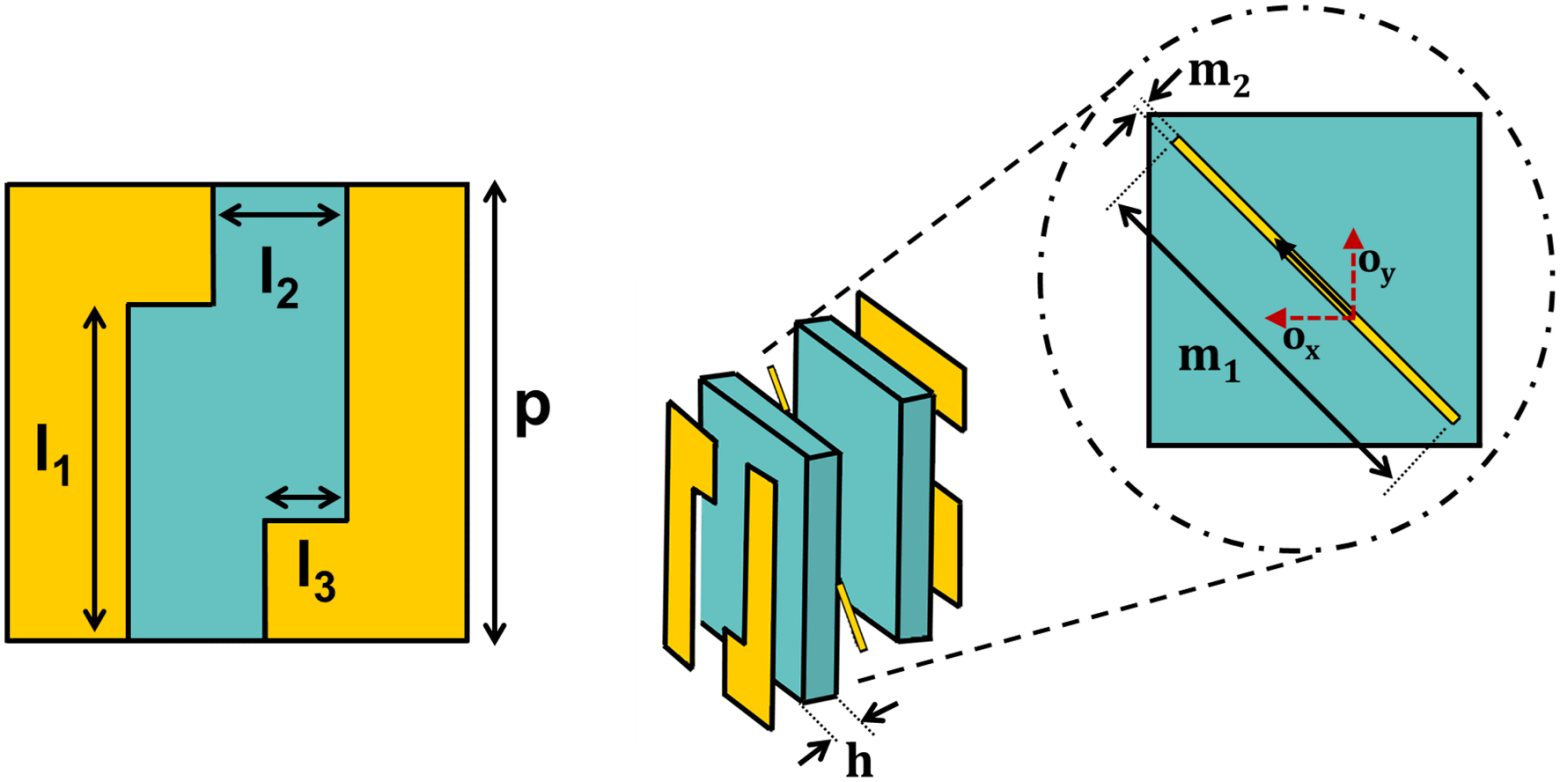
Geometry of the unit cell: $p=8.35$ mm, ${l_1}=6.15$ mm, ${l_2}=2.43$ mm, ${l_3}=1.52$ mm, $h=1.16$ mm, ${m_1}=10$ mm, ${m_2}=0.25$ mm Rogers 4725JXR dielectric (${\epsilon }=2.64$, copper cladding= 35 ${\mu }$m).

Figure 3 illustrates the unit-cell design comprising of three metallic layers sandwiched together by two dielectric substrates and a prepreg, forming a multi-layered structure. The top and bottom metallic layers are $90^{\circ }$ rotated and mirrored versions of each other and resemble strip-like unit cell design that is similar to^31^. The middle layer constitutes a metallic cut-wire resonator which is rotated at $45^{\circ }$ so that an incident wave can cause a dipolar oscillation along its main axis whose orthogonal components (${o_x}$ and ${o_y}$) can interact and thus contribute towards the polarization conversion phenomena^37^. Furthermore, two-fold rotational symmetry of the unit cell breaks symmetry in propagation direction thereby ensuring (T_xx_ =T_yy_ and T_yx_ $\ne$ T_xy_). These are the two conditions which are necessary for asymmetric transmission to occur for linearly polarized waves^38,39^.

The working mechanism of the three metallic layers is explained as follows:

1. The top metallic layer, from the view-point of an array resembles a vertical metallic grating that fundamentally acts as a polarization selector for TM (x-polarization) waves. In other words, an electric field oriented in x-direction will suffer no hindrance by the vertical grating and will pass through. An incident y polarized wave, on the other hand will suffer high reflection since its electric field would be tangential to vertical metal gratings.
2. The bottom metal layer resembles a horizontal grating and therefore acts as a polarization selector for TE (y-polarization) waves. An incident y-polarized wave is selected while an x-polarization is completely reflected. The top and bottom layers, together act as orthogonal gratings that aid in the cross polarization conversion (CPC) phenomenon.
3. The middle metal acts as partially reflective/transmittive layer that interacts with both types of polarizations and aids in producing internal reflections in the structure. The middle layer together with top and bottom gratings results in Fabry-Perot-like cavity that is explained later in the subsection “Fabry-Perot-Like Resonance in the Tri-layered Structure”.

**Figure 4:**
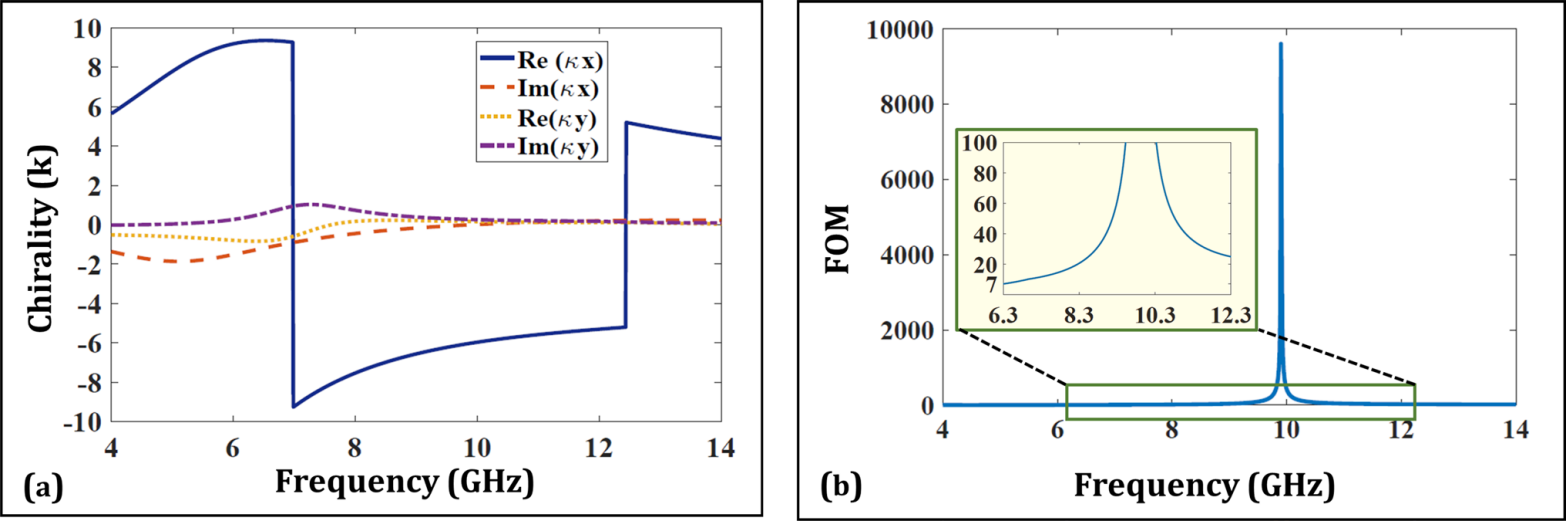
(**a**) Plotted chirality parameter for the structure. (**b**) Plotted FOM on chirality parameter.

The phenomenon of AT stems from the electromagnetic cross coupling that occurs within a structure and can be mathematically expressed by a parameter known as chirality ($\kappa$)^40^. Since, the proposed structure lacks planes of mirror symmetry, it presents a chiral media to the incident EM fields which is governed by the following constitutive relation^41^:

1 $$\begin{aligned} \begin{bmatrix} {D}\\ {B} \end{bmatrix}= \begin{bmatrix} {\epsilon } &{} {-\frac{j{\kappa }}{c}}\\ \frac{j{\kappa }}{c} &{} {\mu } \end{bmatrix} \begin{bmatrix} {E}\\ {H} \end{bmatrix} \end{aligned}$$

($\kappa$), in the above equation, quantifies the extent of electromagnetic cross coupling in a structure and can be extracted through the following parameter retrieval equations^14,42–44^:

2 $$\begin{aligned} Re({\kappa }^{x,y})=\frac{arg(T_{+}^{x,y})-arg(T_{-}^{x,y})}{2{k_0}h} \end{aligned}$$

3 $$\begin{aligned} Im({\kappa }^{x,y})=\frac{ln\mid T_{-}^{x,y}\mid -ln\mid T_{+}^{x,y}\mid }{2{k_0}h} \end{aligned}$$

where, *h* and $k_0$ are the structure thickness and wave number respectively while $T_{\pm }^{x} = T_{xx} \mp jT_{yx}$ and $T_{\pm }^{y} = T_{yy} \pm jT_{xy}$. Figure 4 plots this parameter for the proposed multi-layered chiral structure. It can be observed that the real part of chirality remains greater than 5 in the operating band of interest which denotes higher cross coupling of EM fields. In order to further quantify the quality of chirality in the structure, its figure of merit (FOM = Re($\kappa$) / Im($\kappa$)) is also plotted that shows significant value of greater than 7 in the whole band^45^.

### Analysis of scattering parameters

Asymmetric transmission can also be expressed as the difference between transmission coefficients $T_{yx}$ and $T_{xy}$ as given below^27^:

4 $$\begin{aligned} {\Delta }^{x} = {\mid T_{yx} \mid }^2 - {\mid T_{xy} \mid }^2 = - {\Delta }^{y} \end{aligned}$$

**Figure 5:**
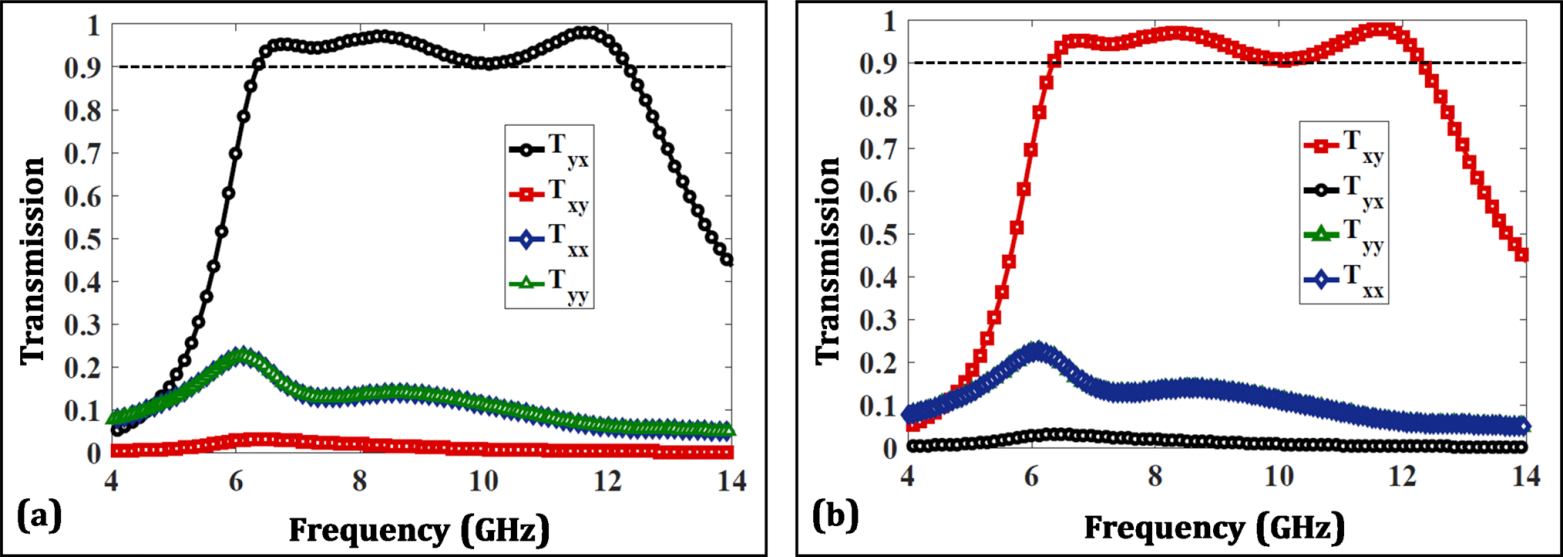
Transmission parameters (**a**) -z-direction (**b**) +z-direction.

It is evident from the above equation that the magnitude of AT is directly influenced by the above difference. The transmission behavior is further elaborated through the well-known Jones Calculus as below:

5 $$\begin{aligned} \begin{bmatrix} T_x\\ T_y \end{bmatrix} = \begin{bmatrix} T_{xx} &{} T_{xy}\\ T_{yx} &{} T_{yy} \end{bmatrix}^{\pm {z}} \begin{bmatrix} I_x\\ I_y \end{bmatrix} = T^{\pm {z}} \begin{bmatrix} I_x\\ I_y \end{bmatrix} \end{aligned}$$

where $I_{x}$ and $I_{y}$ denote the incident EM wave while $T_{x}$ and $T_{y}$ represent the transmitted wave after interacting with the matrix. Figure 5 illustrates the transmission parameters for the proposed multi-layered chiral structure simulated in CST Microwave Studio. It can be seen that when the structure is excited from -z-direction, it passes an incident x-polarized wave as converted y-polarized one while a y-polarized wave impinging in same direction is transmitted negligibly. This is represented by the coefficient $T_{yx}$ that also reveals a broadband response of 6.3-12.3 GHz above 0.9 magnitude constituting a bandwidth of 64% and exhibiting three peaks reaching 0.95, 0.97 and 0.98 at 6.7, 8.3 and 11.6 GHz, respectively. $T_{xy}, T_{xx}=T_{yy}$ on the other hand remain negligible in the operating band resulting in enhanced AT.

Moreover, it can be observed in Fig. 5b that the two cross polarized coefficients swap their magnitudes when the structure is excited in the +z-direction. For instance, at 11.6 GHz, the T matrix is:

6 $$\begin{aligned} T_{11.6GHz}= \begin{bmatrix} 0.067 &{} 0.004\\ 0.980 &{} 0.067 \end{bmatrix}^{-z}\approx {\begin{bmatrix} 0 &{} 0\\ 1 &{} 0 \end{bmatrix}_{ideal}^{-z}} \end{aligned}$$

**Figure 6:**
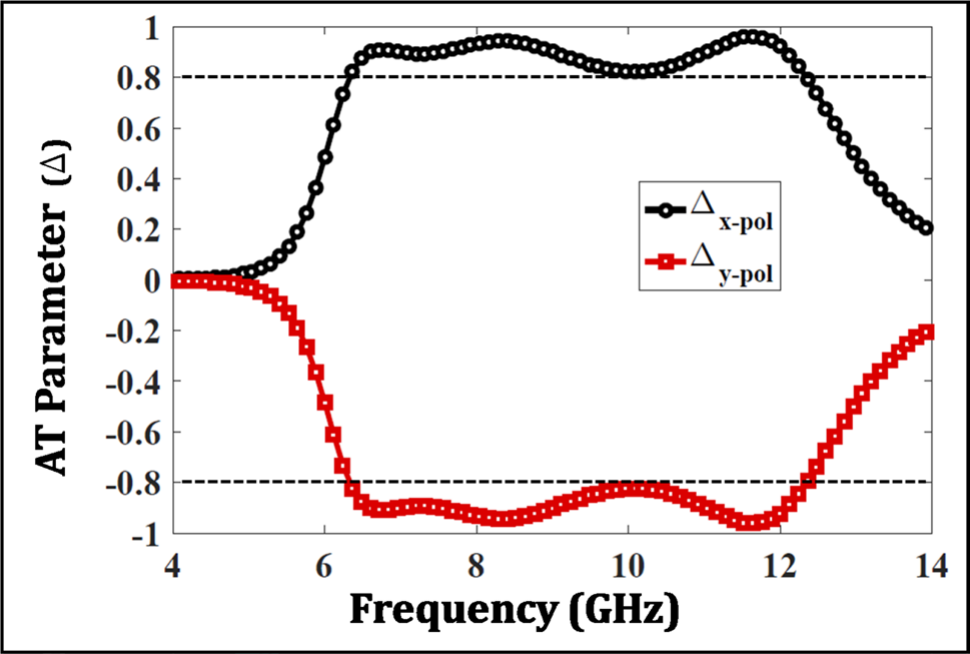
Asymmetric transmission parameter.

The above transmission coefficient values are fairly close to an ideal AT confirming an efficient design. Figure 6 plots the AT parameter extracted from the transmission parameters and assures a magnitude of above 80% in the operating band of interest.

**Figure 7:**
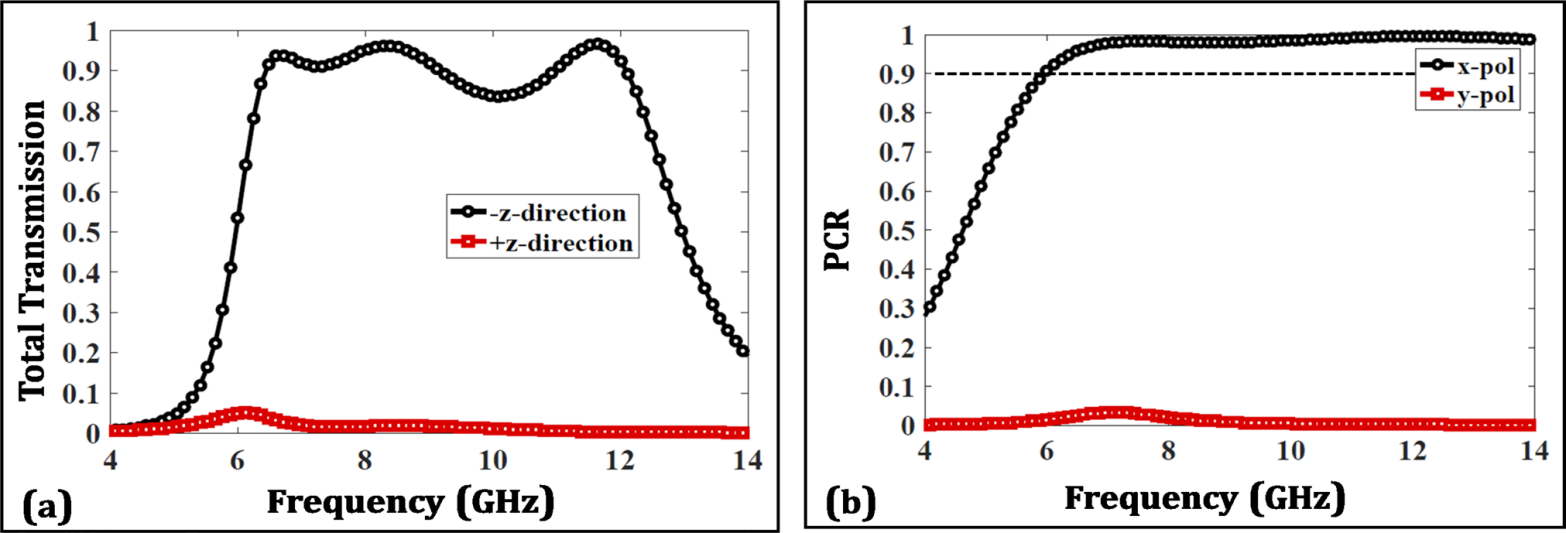
(**a**) Total transmission. (**b**) Polarization conversion ratio (PCR).

To further characterize the AT performance , Fig. 7 illustrates the total transmission in both directions through the polarization conversion ratio (PCR), that is given as:

7 $$\begin{aligned} Total Transmission= & {} {\mid T_{yx} \mid }^2 + {\mid T_{xx} \mid }^2 \end{aligned}$$

8 $$\begin{aligned} PCR= & {} \frac{{\mid T_{yx} \mid }^2}{{\mid T_{yx} \mid }^2 + {\mid T_{xx} \mid }^2} = \frac{{\mid T_{yx} \mid }^2}{TotalTransmission} \end{aligned}$$

The total transmission for an x-polarized wave in -z-direction remains greater than 80% in the whole range with peaks of 0.938, 0.961 and 0.966 at 6.7, 8.3 and 11.6 GHz, respectively. On the other hand, the transmission remains fairly low in + z-direction. Likewise, the PCR which demonstrates the percentage of transmitted EM wave that undergoes cross-polarization conversion also remains close to unity in a wide operating band as shown in Fig. 7b.

**Figure 8:**
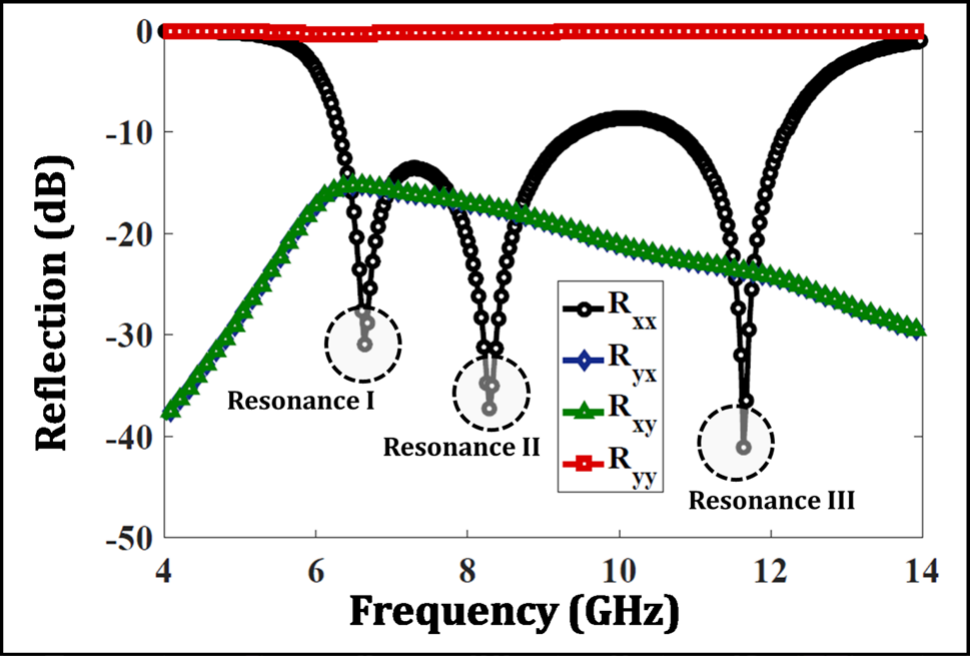
Reflection parameters with identification of three plasmonic resonances.

Asymmetric transmission is a resonant phenomenon and is therefore greatly influenced by reflection parameters of the chiral structure as illustrated in Fig. 8. $R_{yy}$ is around 0dB in the whole range indicating that a y-polarized wave illuminated in -z-direction is significantly reflected. On the other hand, $R_{xx}$ depicts three resonances at 6.6, 8.3 and 11.6 GHz, respectively. The broadband transmission behavior of the metasurface is in fact due to the close proximity of the three plasmonic resonances with one another. In other words, at these resonances, the structure presents a surface impedance to an x-polarized wave which is closer to the free space impedance and therefore results in efficient transmission.

### Fabry-perot like resonance in the tri-layered structure

**Figure 9:**
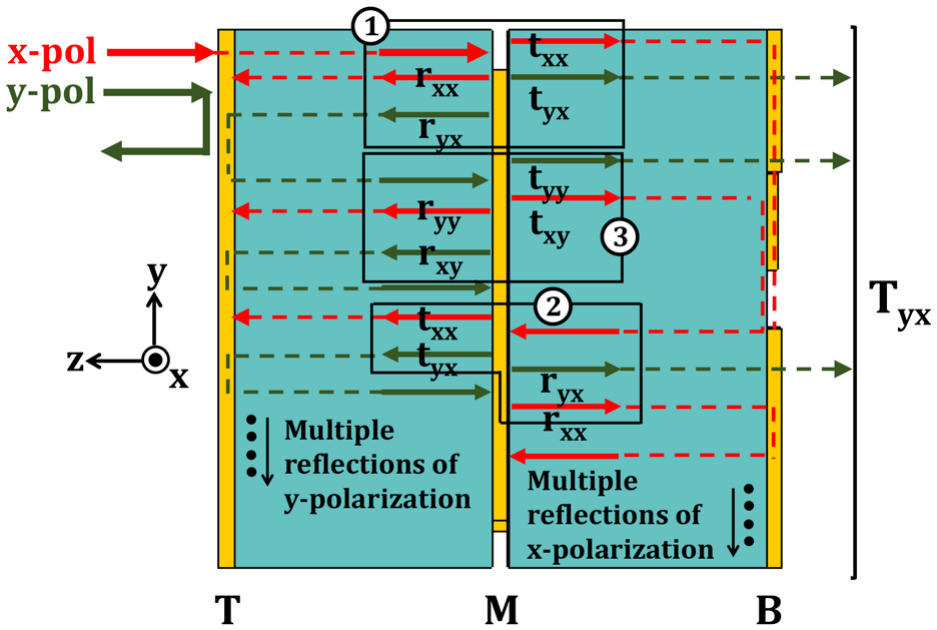
Multiple reflections occurring in multi-layered structure, resembling a Fabry-Perot Cavity.

Better understanding of the tri-layered structure can be achieved through Fig. 9. The top and bottom layers (T & B), being similar to orthogonal metallic gratings, act as x and y-polarization selectors. An incident y-polarization in -z-direction suffers reflection while an x-polarized wave is selected by layer T. The x-polarized wave then passes through layer T and moves on to interact with the middle layer (M) which breaks the wave into four parts ($t_{xx}$, $t_{yx}$, $r_{xx}$, $r_{yx}$) as shown in the labeled region 1. Out of the two transmitted waves $t_{yx}$ is selected by layer B thus contributing towards the resultant y-polarization at the output. $t_{xx}$, on the other hand, suffers reflection by B and on return also interacts with middle layer to be broken into four parts as shown in region 2. The reflection $r_{yx}$ from region 1 suffers another reflection from the layer T and similarly, is again broken into its constituents by the middle layer (region 3). Continuing the same analysis it can be seen that selectors T and B along with middle layer M cause multiple reflections of y-polarization in the MT region and that of x-polarization in MB region. These multiple reflections resemble a Fabry-Perot cavity^46^ that, in turn, results in enhanced peaks of transmission.

### Angular stability of designed metasurface

#### Response to oblique incidences

**Figure 10:**
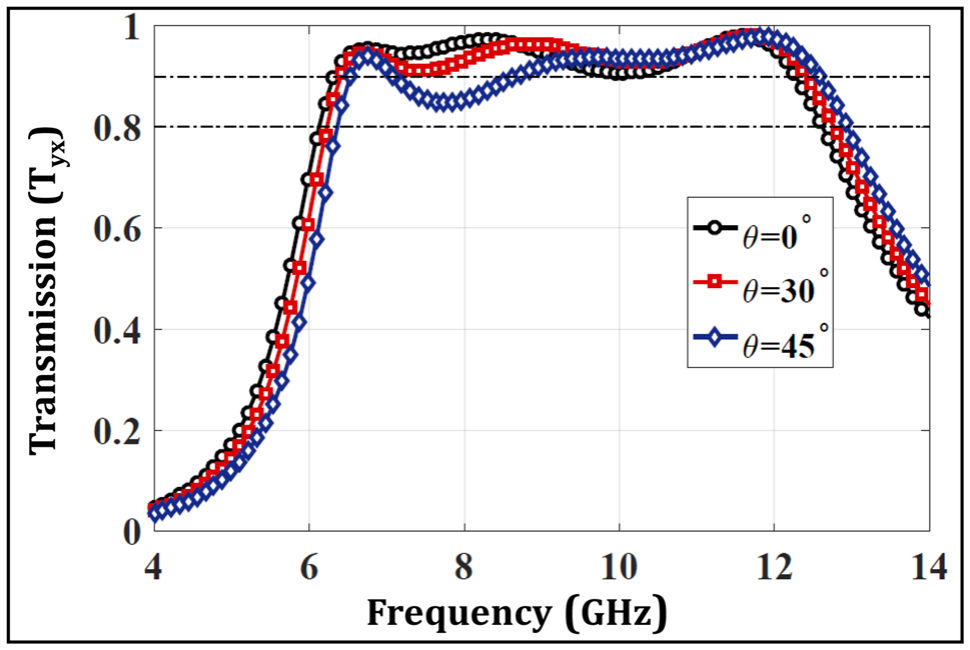
Transmission behavior with respect to different incident angles for proposed structure.

**Figure 11:**
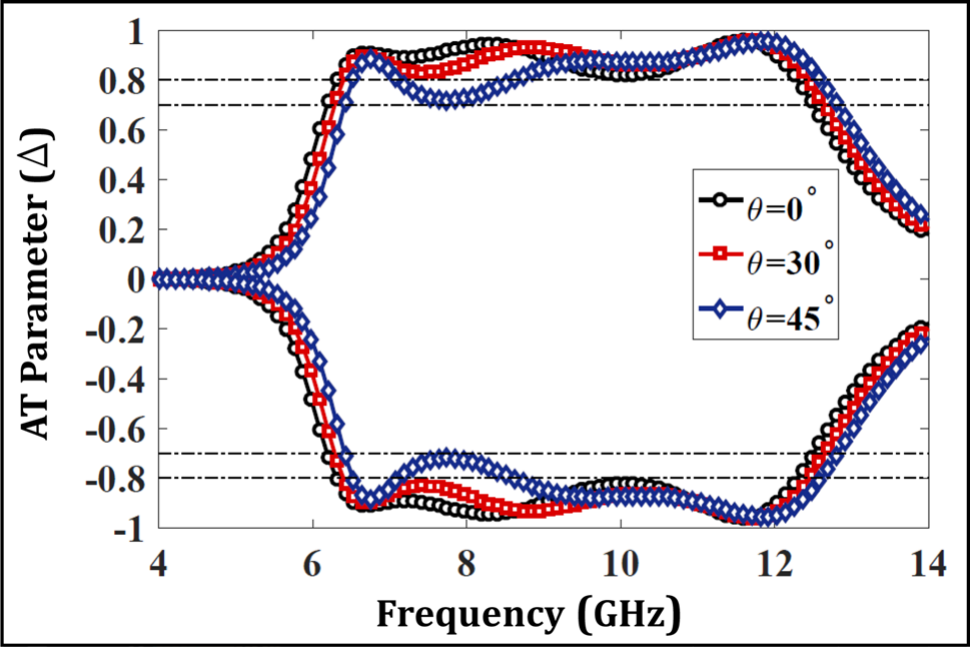
Variation of AT with incident angles for the proposed structure.

AT devices, when employed in practical environments should offer robust performance for varying incident angles. In order to analyze this, the transmission response has been plotted against multiple oblique incidences ranging from $0^{\circ }$ to $45^{\circ }$ in Fig. 10. It is observable that the transmission response maintains a level of greater than 80% against the varying incident angles indicating a robust performance in the whole frequency range of interest. Likewise, the AT parameter at the same angles is shown in Fig. 11 that shows a magnitude greater than 70% indicating an angularly stable performance of the proposed AT device.

#### Surface impedance mismatch at oblique incidences

This subsection provides insights on the dependence of AT phenomenon on the concept of surface impedance mismatch. As the incident angle increases, the resonances in the structure become weaker and result in magnitude drop of transmission with increasing $\theta$. This is in fact caused by the varying surface impedance ($Z_0$) that is strongly associated with the incident angle. More specifically, for TM (x-polarization), $Z_0$ is inversely proportional to $\theta$ while it is directly proportional in case of TE (y-polarization) as shown in following two equations (4) and (5)^47^:

9 $$\begin{aligned} {Z_{0}}^{TM}= & {} {{\eta }_0}{\cos {\theta }} \end{aligned}$$

10 $$\begin{aligned} {Z_{0}}^{TE}= & {} \frac{{\eta }_0}{\cos {\theta }} \end{aligned}$$

where, ${\eta }_0$ is the free space impedance.

**Figure 12:**
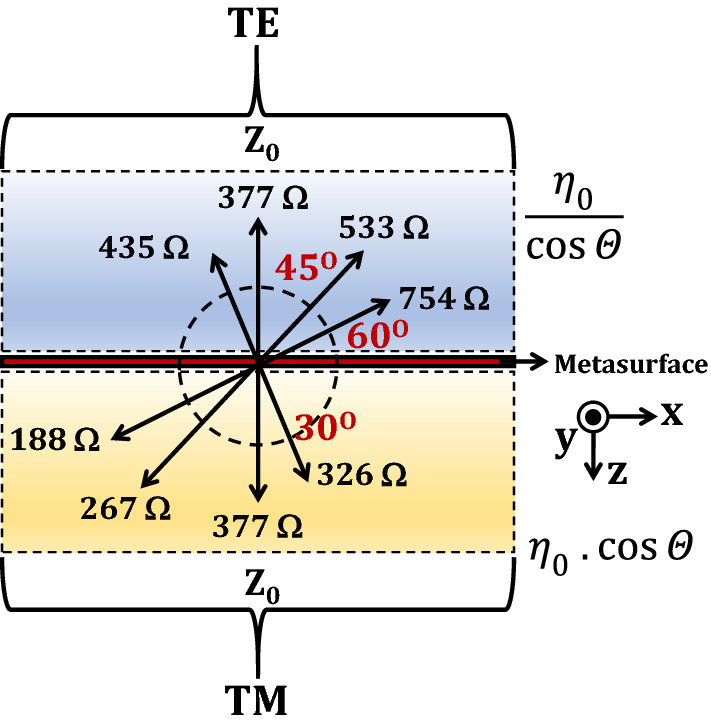
Sensitivity of asymmetric transmission at oblique incidences illustrated through the concept of increasing impedance mismatch at higher angles.

The role of surface impedance in case of an ideal asymmetric transmission is illustrated in Fig. 12. At normal incidence (${\theta }=0^{\circ }$), an incident TM wave is fully transmitted as a TE wave, hence the impedance at both the floquet ports of a metasurface is equal to the free space impedance of 377 ohms. However, when the incident angle increases, TM ports suffers a positive mismatch (e.g. ${\eta }_0-Z_0=377-188= +189 \Omega$) while TE ports suffers a negative mismatch (e.g. ${\eta }_0-Z_0=377-754= -377 \Omega$), thus increasing the overall impedance mismatch ($189-(-377)=566 \Omega$) between the two ports. The increasing mismatch at higher incident angles due to two different ports (TE & TM) inherently makes the magnitude of asymmetric transmission sensitive to oblique incidences and therefore it is challenging to develop an angularly stable AT device.

#### Avoidance of grating lobes at higher incidence angles

**Figure 13:**
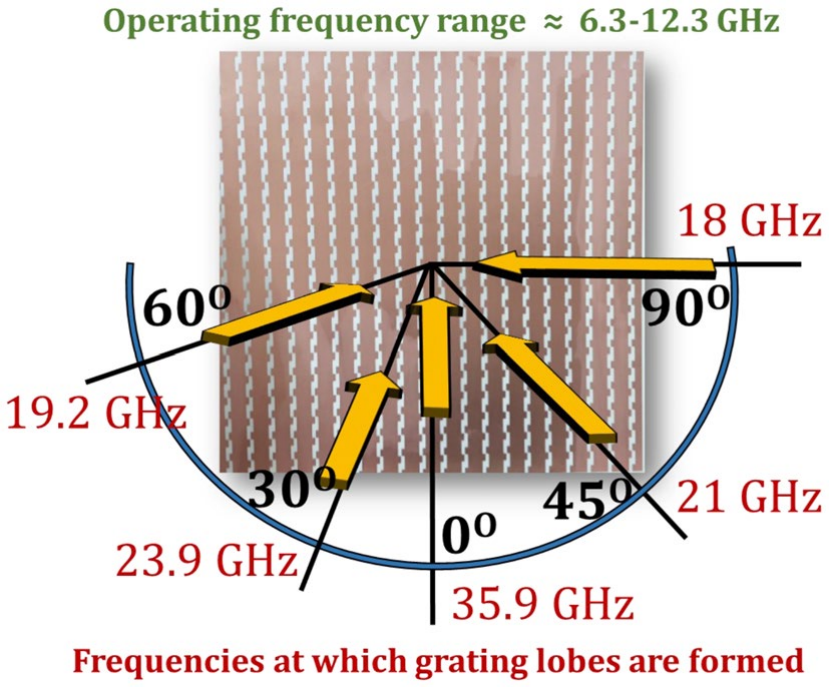
Predicted frequencies of grating lobes.

Study of FSS theory reveals that the deterioration in angular performance of a metasurface is caused by interference of secondary main lobes formed at higher angles of incidence^48,49^. The phenomena of such unwanted beams, better known as grating lobes, occurs when the periodicity (p) of an array becomes electrically large. In order to avoid this, metasurface periodicity should be electrically small in accordance with following relation:

11 $$\begin{aligned} p<{\frac{\lambda }{1+\sin {\theta }}} \end{aligned}$$

where, $\lambda$ is the wavelength at highest operating frequency. The required value of p for an angularly stable performance is calculated to be less than 12.1 mm which corresponds to $0.5\lambda$ ($p<{\frac{24.3 mm}{1+\sin {90^{\circ }}}}$). The periodicity of reported metasurface with $p=8.3 mm$ ($0.3\lambda$) is significantly smaller and hence effectively avoids interference caused by the grating lobes. Furthermore, using the relation given in (7), Fig.  13 depicts the predicted frequencies at which grating lobes are formed against different incident angles.

12 $$\begin{aligned} {f_{G.L}}={\frac{c}{p(1+\sin {\theta })}} \end{aligned}$$

where, c is the speed of light^50^. It is evident that for all the angles of incidence, grating lobes are formed at frequencies that are far away from the operating band of interest, thus making the reported metasurface angularly stable.

To further clarify the concept, consider a design that functions exactly the same as the reported design i.e. manifesting AT in the frequency range 6.3 to 12.3 GHz, however, its periodicity is larger than $0.5\lambda$ e.g. 30 mm. The grating lobes at 30$^{\circ }$ in this case would form at 6.6 GHz which lies in the operating range and therefore would result in a deteriorated performance at oblique incidences. Thus a miniaturized unit cell is necessary for angular stability. Furthermore, the stability at higher angles of incidence is dependent upon the stability of the resonances in the structure and since AT is a resonant phenomenon, it can therefore be deteriorated if grating lobes are not avoided at higher angles.

### Experimental validation

**Figure 14:**
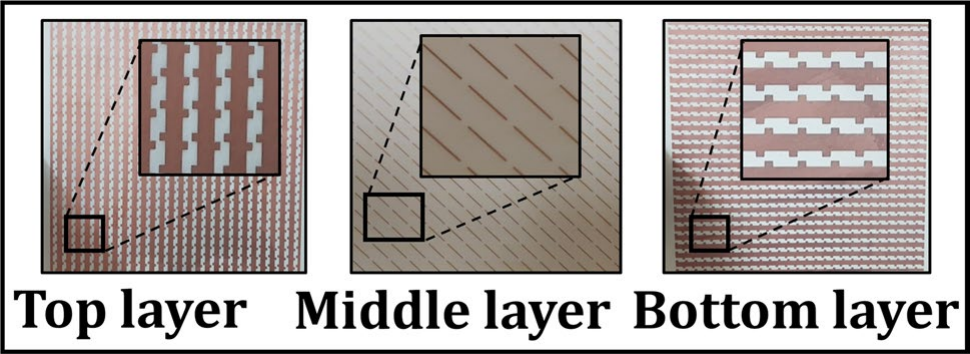
Fabricated prototype of the multi-layered design.

**Figure 15:**
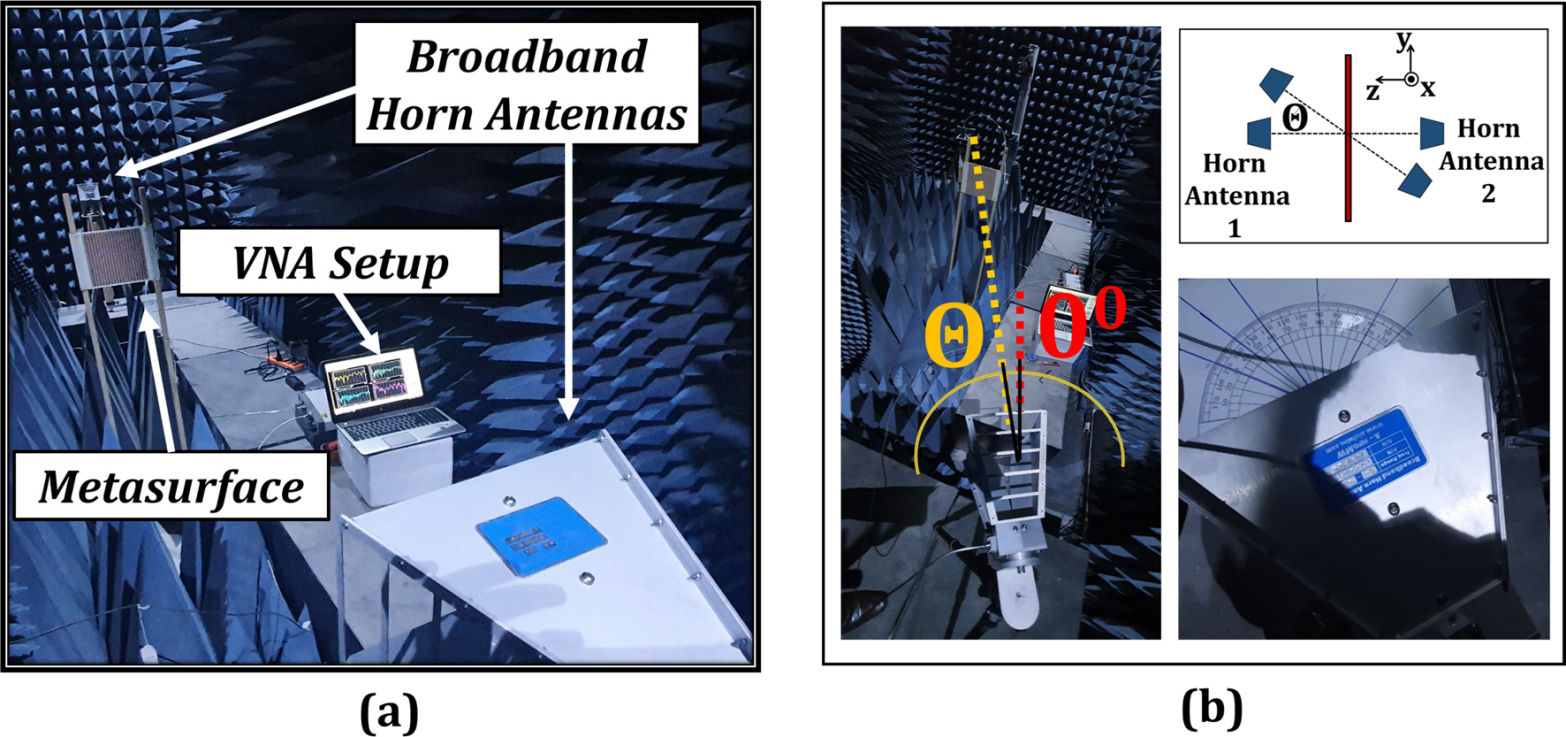
(**a**) Measurement setup (**b**) Measurement at oblique incidences.

To test the reported multi-layered chiral metasurface, an array of $27\times 27$ unit cells with an electrical size of ${7\lambda \times 7\lambda \times 0.07\lambda }$ was fabricated using PCB fabrication technology (Fig. 14). The two Rogers substrates were thermally fused using a thin FR4 prepreg. The measurement setup typically consists of two broadband horn antennas with the metasurface between them which are connected to a VNA through coaxial cables as shown in Fig. 15. The antennas are distanced 4 meters from one another to mitigate for the near field effects and Anritsu-MS46122B VNA is used. Moreover, in order to measure the performance at oblique incidences, the antennas were manually rotated around the central axis of metasurface as indicated in Fig. 15(b).

**Figure 16:**
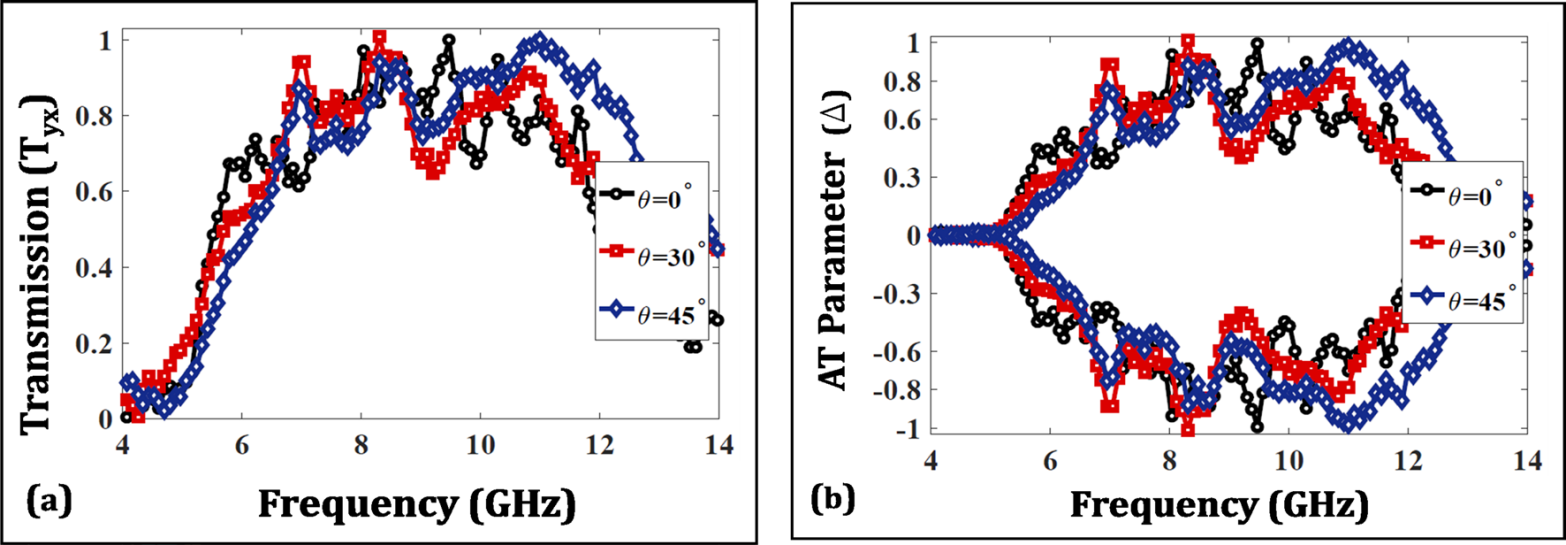
Measured results (**a**) Transmission parameter ($T_{yx}$) (**b**) AT parameter.

The measured curves for $T_{yx}$ and AT parameter ($\Delta$) for the fabricated prototype are shown in Fig. 16 and show a similar trend to the simulated results. Moreover, the metasurface maintains robustness against oblique incidences ranging from $0^{\circ }$ to $45^{\circ }$ hence validating an angularly stable performance. The discrepancies from simulations stem from the handling inaccuracies and lack of focusing of EM waves ( due to unavailability of dielectric lens). Furthermore, multiple wave reflections also cause fluctuations, which can be minimized using a VNA with time gating functionality to monitor the first echo only. A dedicated metasurface measurement setup can further enhance the match between the measurements and simulations.

## Discussion

Table 1. shows a comparison of presented metasurface with a few recent works that are relevant in the microwave regime on the merits of operational percentage bandwidth and angular performance^32^ reports a broadband AT operation that maintains efficiency up to 30$^{\circ }$. However, its performance degrades at higher oblique incidences. The designs in^35^ and^36^ manifest angular performance up to 60$^{\circ }$ and 40$^{\circ }$ in bi-layered and tri-layered configurations, respectively, however, they are limited to a narrow band operation. Furthermore, the recent publication by the authors^31^ although achieves an angularly robust performance up to 60$^{\circ }$, however is limited to a bandwidth of 18% that has been improved with a tri-layered variant of the design which ensures a ultra-wide operational bandwidth of 64% with a wide angle performance up to 45$^{\circ }$.

Operational bandwidth, in addition to angular stability is a critical parameter for practical applications. Electromagnetic systems are usually prone to two types of variations; spatial variations (related to space e.g. angle of incidence) and temporal variations (related to frequency). Angular robustness of a device caters for the former type of instability while robustness to frequency drift caters for the latter. More importantly, the frequency signals in a practical environment are not necessarily single tones and have a finite bandwidth. To that end, the presented work is robust to frequency variations owing to its wide operating bandwidth of 64%. This feature makes it ideal for practical applications.

**Table 1:** Comparison with recently published works.

Ref	Year	Configuration	Max. BW at normal incidence (AT above 0.8)	Performance at oblique incidences (AT above 0.7)
^32^	2018	Bi-layered	15%	30$^{\circ }$
^35^	2020	Bi-layered	2%	60$^{\circ }$
^36^	2020	Tri-layered	3%	40$^{\circ }$
^31^	2020	Bi-layered	18%	60$^{\circ }$
^21^	2020	Bi-layered	18% approx.	60$^{\circ }$
This Work	2021	Tri-layered	64%	45$^{\circ }$

In conclusion, the work presented in this report firstly introduces AT operation in analogy with logic gates circuit which may be extended to more complex optical communications systems. The proposed tri-layered chiral metasurface, benefiting from Fabry Perot-like resonances achieves an ultra-broadband operational bandwidth of 64% (6.3 - 12.3 GHz) while maintaining a transmission efficiency of more than 80% and angular stability of up to 45$^{\circ }$. Moreover, the susceptibility of AT to higher incident angles is described in terms of surface impedance mismatch between TE and TM ports. Mitigation of grating lobes through design of a miniaturized unit cell is also explained. The performance has been validated through measurements thereby providing designers a complete design methodology to produce angularly robust devices for modern optical communication and radar systems.

## Methods

Free space method was used to measure the transmission parameters of the fabricated metasurface that is governed by the following expression:

13 $$\begin{aligned} S_{21}=\frac{{S^{metamaterial}_{21}}-{S^{MetallicSheet}_{21}}}{{S^{airspace}_{21}}-{S^{MetallicSheet}_{21}}} e^{-j{k_0}d} \end{aligned}$$

where, measurements are done with a metallic sheet, air and metasurface between the antennas to get $S^{MetallicSheet}_{21}$, $S^{airspace}_{21}$ and $S^{metamaterial}_{21}$, respectively^51^.
